# Gene Expression and Methylation Pattern in HRK Apoptotic Gene in Myelodysplastic Syndrome

**Published:** 2016-06-19

**Authors:** Farhad Zaker, Naser Amirizadeh, Nahid Nasiri, Seyed Mohsen Razavi, Ladan Teimoori-Toolabi, Marjan Yaghmaie, Roya Mehrasa

**Affiliations:** 1 Cellular and Molecular Research Center, Iran University of Medical Sciences, Tehran, Iran.; 2 Department of Hematology, School of Allied Medicine, Iran University of Medical Sciences, Tehran, Iran.; 3 Blood transfusion research center, High institute for Education and Research in Transfusion Medicine, Tehran, Iran.; 4 Hematology and Oncology Department, Firoozgar Hospital, Iran University of Medical Sciences, Tehran, Iran.; 5 Molecular Medicine Department, Biotechnology Research Center, Pasteur Institute of Iran, Tehran, Iran.; 6 Hematology, Oncology and Stem cell Transplantation Research Center, Tehran University of Medical Sciences, Tehran, Iran.

**Keywords:** High Resolution Melting (HRM), methylation, myelodysplastic syndrome, *HRK*

## Abstract

Myelodysplastic syndromes (MDSs) are a clonal bone marrow (BM) disease characterized by ineffective hematopoiesis, dysplastic maturation and progression to acute myeloid leukemia (AML). Methylation silencing of *HRK *has been found in several human malignancies. In this study, we explored the association of *HRK* methylation status with its expression, clinical parameters and MDS subtypes in MDS patients. To study the methylation status of *HRK* gene, we applied Methylation Sensitive-High Resolution Melting Curve Analysis (MS-HRM) in MDS patients, as well as healthy controls and EpiTect®PCR Control DNA. Real time RT-PCR was used for gene expression analysis. Methylation frequency in promoter region of *HRK *in patient samples was 20.37%. Methylation of *HRK *was significantly related to transcriptional downregulation (P=0.023). The difference in frequency of hypermethylated *HRK* gene was significant between good (10%) and poor (71.42%) cytogenetic risk groups (P= 0.001), advanced stage MDS patients (66.66%) in comparison with early stage MDS patients (2.56%) (P= 0.00), higher- risk MDS group (61.53%) and lower- risk MDS group (7.31%) (P= 0.00). *HRK* hypermethylation was associated with advanced- stage MDS and downregulation of *HRK* gene may play a role in the progression of MDS.

Myelodysplastic syndromes (MDSs) constit-ute a heterogeneous group of clonal bone marrow disorders characterized by ineffective hematopoiesis with subsequent cytopenia, dysplastic maturation of blood cells and potential transformation to acute myeloid leukemia (AML) (1, 2). Although the pathogenic mechanisms of cytopenia and disease-progression to secondary AML in MDS have been poorly understood, apoptotic deregulation is considered as one of the underlying mechanisms (3). Methylation of promoter CpG sites plays a major role in gene silencing (4). Restoration of gene function and growth control in MDS and AML by demethylating agents such as 5-aza-2_-deoxycytidine and 5-azacytidine represent causative role of aberrant hypermethylation in the disease (5, 6). Efficacy of demethylating agents may result from induction of differentiation, cytotoxicity, or changes in apoptosis (7-9). It has been shown that dysregulation of cellular epigenetic machinery, particularly aberrant methylation of DNA, is a key factor contributing to the pathogenesis of MDS and may trigger leukemic transformation (10, 11).

Owing to the importance of apoptotic pathway in MDS, we decided to examine the methylation of Harakiri* (HRK)* gene, which is involved in the apoptosis pathway. Although the *HRK* methylation status has been extensively studied in cancers, there are no reports in this regard in MDS. HRK is a proapoptotic mitochondrial member of Bcl-2 family, which promotes apoptosis through the endogenous, mitochondrial pathway. This protein interacts with survival-promoting proteins Bcl-2/Bcl-xL/MCL1 or P32 and facilitates the release of cytochrome C, formation of apoptosome and activation of the caspase cascade (12-14). HRK lacks BH1, BH2 and BH4 domains but shares the BH3 region. The proapoptotic activity of HRK is mediated by BH3 domain, which is critical for the association between BH3-only protein and anti-apoptotic proteins (12). *HRK* is expressed in normal tissues but its decreased expression has been reported following promoter methylation in many cancers such as melanoma, prostate cancer and astrocytic tumors (14, 15). Moreover, transcrip-tional repression of *HRK *by aberrant methylation*, *especially in combination with loss of hetero-zygosity (LOH), is a potential step in modulation of programed cellular death (14). Since suppression of *HRK* occurs in cancers, it may play an important role in the development and progression of human tumors (14). Considering the importance of this gene in the intrinsic apoptotic pathway, it seems rational that any defects in its expression may play an effective role in oncogenesis.

This study aimed to determine whether DNA methylation affects *HRK* expression in cells of MDS patients. We further investigated the correlation of aberrant methylation and expression of *HRK* with clinicopathological parameters at different subgroups of disease. No study has been performed on Iranian-MDS patients in this respect, so far.

## Materials and methods

**Patients and samples**

Sixty MDS patients (de novo or only treated with transfusion) referred to Shariati and Firouzgar Hospitals (Tehran, Iran) were included in this study after Informed consent was obtained. Six patients were excluded due to unavailability of some clinical data. To evaluate the clinical impact of *HRK* methylation, we have investigated the following variables: age, sex, white blood cell (WBC), absolute neutrophil count (ANC), hemoglobin (Hb) level, platelet (Plt) count, bone marrow blasts, LDH and SF levels, cytogenetics, World Health Organization (WHO) classification and revised international prognostic scoring system (IPSS-R). Clinical features of the patients are listed in Table 1. As controls, DNA from 20 healthy volunteers, with median age of 62.45 years (range, 45-83 years), as well as EpiTect®PCR control DNA (Qiagen, Hilden, Germany) were used in experiments. Patients were classified in accor-dance with 2008 version of WHO classification of MDS. The study was approved by the Ethics Committee of Iran University of Medical Sciences.

**Conventional cytogenetic analysis**

Conventional cytogenetic analysis was perfor-

med for 54 patients. Bone marrow cells were cultured for 24 h. Finally, the cells were fixed with methanol/acetic acid and their karyotypes were investigated on banded metaphases prepared by conventional GTG banding technique. Chromo-some abnormalities were characterized based on the international system for human cytogenetic nomen-clature (16).

**Table 1:** Association between *HRK* methylation and clinicopathological variables in MDS patients.

**Characteristics**	***HRK*** **Methylated (11)**		***HRK *** **Unmethylated (43)**		P-value
	Median (range)	n (%)	Median (range)	n (%)	
**Age** (Years)	59 (23-75)		62.18 (38-90)		0.525
**Sex ** Male Female		- 4 (13.8%) 7(28%)		- 25 (86.2%) 18 (72%)	0.196
**WBC** (×10^9^/L)	3.1(1.2-5)		5.4 (1.5-1.38)		0.00
**ANC** (×10^9^/L)	1.3(0.29-3.2)		3 (0.59-8.4)		0.00
**Hb** (g/L)	9.58 (6.8-14.1)		9.61 (5.6-14.8)		0.963
**Platelets** (×10^9^/L)	80.36 (30-141)		149.6 (7-752)		0.105
**SF **(ng/ml)	407.73 (134-690)		380.89 (2.9-1600)		0.805
**LDH **(U/L)	664.11 (385-1886)		345 (99-1022)		0.00
LDH>400U/l LDH<400U/l		10 (45.45%) 1 (3.1%)		12 (54.54%) 31 (96.9%)	0.00
**BM Blast (%)** <5% >5%		1 (2.56%) 10 (66.66%)		38 (97.43%) 5 (33.33%)	0.00
**Karyotype(IPSS-R) ** Very Good Good Intermediate Poor Very poor		- 0 (0%) 4 (11.11%) 2 (25%) 2 (66.66%) 3 (100%)		- 4 (100%) 32 (88.88%) 6 (75%) 1 (33.33%) 0 (0%)	0.001
**IPSS-R ** Very low Low Int High Very high		- 1 (5%) 1 (7.7%) 1 (12.5%) 1 (25%) 7 (77.77%)		- 19 (95%) 12 (92.3%) 7 (87.5%) 3 (75%) 2 (22.22%)	0.00
**WHO classification** RA RT RCMD RAEB-1 RAEB-2 -5q		- 1 (5%) 0 (0%) 0 (0%) 3 (75%) 7 (63.63%) 0 (0%)		- 19(95%) 6 (100%) 10 (100%) 1 (25%) 4 (36.36%) 3 (100%)	0.00

WBC: white blood cell count; ANC: absolute neutrophil count; Hb: hemoglobin; Plt, platelet; SF: serum ferritin; LDH: lactate dehydrogenase; IPSS-R: international prognostic scoring system (revised); WHO: world health organization; RA: refractory anemia; RT: refractory thrombocytopenia; RCMD: refractory anemia with multilineage dysplasia ; RAEB-1: refractory anemia with excess blasts-1; REAB-2: refractory anemia with excess blasts-2; -5q: MDS associated with isolated del (5q-).

**Bisulfite conversion**

Genomic DNA isolation was performed using QIAamp DNA Blood Mini Kit (Qiagen, Hilden, Germany) following manufacturer’s protocols. The amount of DNA extracted was measured by Nanodrop (ND-1000, Thermo scientific, US ). 1000 ng of genomic DNA was converted by EpiTect Bisulfite Kit (Qiagen, Hilden, Germany) as described in manufacturer’s instructions. Modified DNA was suspended in elution buffer and was immediately used or stored at -80 ˚C. Incubation of target DNA with sodium bisulfite results in conversion of unmethylated cytosine residues into uracil, leaving the methylated cytosines unchanged.

**Methylation specific-high resolution melting (MS-HRM)**

MS-HRM was carried out using 20 ng of modified DNA in a 10 μl volume, with 5 μl of EpiTect HRM-PCR Kit (Qiagen, Hilden, Germany) and 2.5 pmole of each primer. The primers for this gene were designed using Meth Primer software (17). Primers should not contain any CpG sites within their sequence. In cases where CpG sites were unavoidable, degenerate bases were used at CpG sites. The primers used for *HRK* were as follows: F: 5′- GAG TTG AAT TTA GGA AAA GGG GAA GG -3′ and R: 5′- CCC CCR AAA ATT AAA AAA AAA ACT ACA AAC-3′ (236 bp). The amplification conditions were as follows: one cycle of 95 °C for 5 minutes, followed by 40 cycles of 95 °C for 10 s, 30 s at 58 ˚C and 20 s at 72 °C. After PCR, the HRM was accomplished as follows: from 56 °C to 99 °C, the temperature was increased by 0.1 °C/2 s. Both amplification and HRM analysis were conducted out in a rotor gene TM 6000 device (Corbett Research, Mortlake, Australia).

**Gene expression analysis**

The expression level of *HRK* was validated by quantitative RT-PCR (qRT-PCR). RNA isolation from mononuclear cells (MNCs) was done by TriPure isolation reagent (Roche Applied Science, Penzberg, Germany), according to manufacturer's instructions. One µg RNA was used per reverse transcription reaction using QuantiTect Reverse Transcription Kit (Qiagen, Hilden, Germany) following manufacturer’s guidelines. cDNA was diluted and stored at -80 °C. Each reaction was performed in a volume of 10 μl containing 5 μl of 2 ×SYBR Green master mix, 2 μl of cDNA and 2.5 pmole of each primer. Glyceraldehyde-3-phosphate dehydrogenase (*GAPDH*) was simultaneously used as the housekeeping gene to normalize the expression of *HRK* gene. Primers used were as follows: for *HRK, *F: 5′- GGCAGGCGGAACTTGTAGGAAC-3′ and R: 5′- TCCAGGCGCTGT-CTTTACTCTCC-3′ (197 bp); for *GAPDH*, F: 5′- CACCAGGGCTGCTTTTAACTCTGGA-3' and R: 5′- CCTTGACGGTGCCATGGAATTTGC-3' (130 bp). The cycling conditions were as follows: 5 min at 95 °C, 40 cycles for 10 s at 95 °C and 30 s at 60 °C (combined annealing/extension step). Each reac-tion was done in triplicate. After gene amplifica-tion, the melting curve analysis was performed. Q-PCR was conducted on rotor gene 6000 device. The relative mRNA expression level of samples was calculated using ∆∆Ct method and was compared with the amount of mRNA in control samples.

**Statistical analyzes**

Statistical analyses were performed using SPSS 16.0 software package (SPSS, Chicago, IL). Mann–Whitney’s U-test and T-test were performed to compare variables between patient and control groups. Kruskal-Wallis test was used to recognize the differences in methylation between subgroups. ANOVA and student’s t-test were performed to evaluate parametric data between different groups. Chi square was utilized as required. For all analyses, the p values were two-tailed, and a p≤0.05 was considered as statistically significant.

## Results

**Patients demography**

54 MDS patients, 29 males and 25 females, were included in the present study. The median age of patients was 61 years (range, 23–90 years). Patient distribution according to WHO classifica-tion was refractory anemia with unilineage dysplasia (RUMD) (n=26, 48.1%), refractory anemia with multilineage dysplasia (RCMD) (n=10, 18.5%), refractory anemia with excess blasts-1 (RAEB-1) (n=4, 7.4%), refractory anemia with excess blasts-2 (RAEB-2) (n=11, 20.4%) and MDS associated with isolated del (5q-) (n=3, 5.6%). Cytogenetic results were observed as follows: normal karyotype in 27 patients (50%) and abnormal karyotype in 27 patients (50%), including; deletion in chromosome 5 in five patients (9.25%), Chromosome 7 abnormalities in 3 (5.55%), trisomy 8 in 4 (7.4%), deletion 20q in 4 (7.4%), monosomy Y in 4 (7.4%), complex cytogenetics in 4 (7.4 %) and other abnormalities in 3 (5.55%) patients. Cytogenetic findings were categorized according to IPSS- R score into very-good (-Y and 11q-), good (Normal karyotype, 5q-, 20q-, 12p- and double including 5q), intermediate (del[7q], +8, +19, i[17q], any other single or double independent clones), poor (-7, inv[3]/t[3q]/del[3q], double including -7/del[7q], Complex [3 abnormalities]) and very poor (complex karyotypes [>3 abnormalities]) (18). IPSS-R was calculated according to the criteria developed by international working group for prognosis of MDS (IWG-PM). Clinical and demographic data are summarized in Table 1.

***HRK*** **mRNA expression in MDS patients**

**Fig 1:**
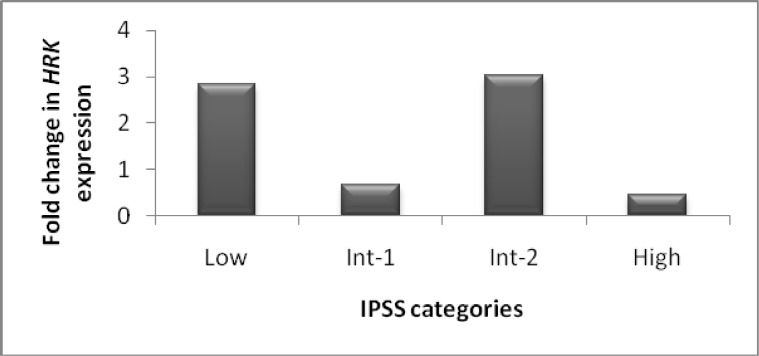
Fold change in *HRK* expression in patients with low, Int-1, Int-2 and high risk MDS (P= 0.015).Decreased gene expression in some patients without methylation represents the role of other mechanisms in *HRK* gene silencing.

QRT-PCR was carried out to specify whether the methylation in transcription start site was associated with suppression of mRNA expression. When the delta Ct of *HRK* was compared between patient and normal groups, the difference was not statistically significant, due to widespread delta Ct between subgroups (P=0.074). But the difference in delta Ct between advanced stage MDS and controls was statistically significant (P=0.025). In comparison of *HRK* expression fold change to DNA methylation level in patient groups, a significant association was observed (P<0.05). The gene expression level was decreased in samples with methylation. To assess the clinical impact of *HRK *expression, we have analyzed the correlation of the gene expression with clinicopathological findings at presentation. Expression of *HRK* was statistically significant between IPSS categories (P= 0.015) (Fig 1). The least fold change was seen in the high risk group. Expression fold change of *HRK* in early stage MDS was higher than that in advanced stage MDS (2.55 vs. 1.5, respectively), but the difference was not statistically significant (P= 0.228). *HRK *expression was significantly higher in the group with LDH<400 U/L than the group with LDH>400 U/L (3.02 vs. 1.23, respectively, P<0.05). There were no significant associations between *HRK* expression fold change and age, gender, hematologic variables and other parameters.

**Fig 2:**
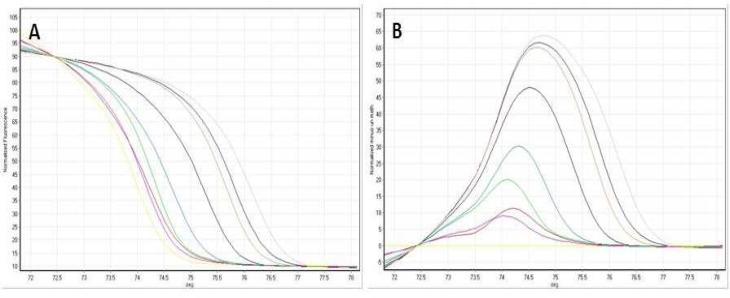
HRM standard curves for HRK. (A) Normalized graph for HRK. (B) Difference plot for the data represented in (**A**). Standards 100% gray line, 90% dark blue line, 80% orange line, 50% black line, 25% blue line, 20% green line, 10% purple line, 0% yellow line, red line for patient sample.

**Fig 3:**
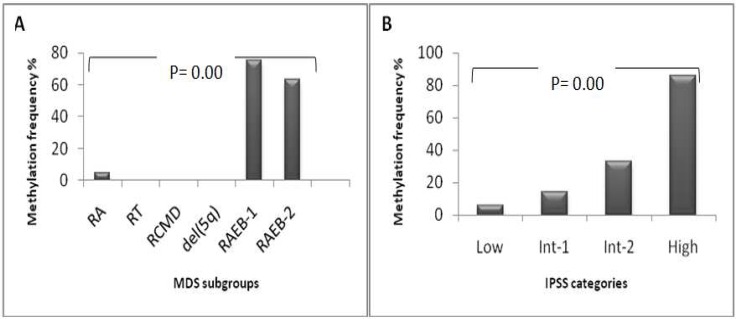
Methylation frequency of the HRK gene in MDS patients. (A) aberrant methylation increased from early stage MDS (RA, RT, RCMD, del[5]) to advanced stage MDS (RAEB-1, RAEB-2) (P= 0.00). (B) aberrant methylation showed a stepwise increase with higher IPSS risk categories (P= 0.00).

**Fig 4:**
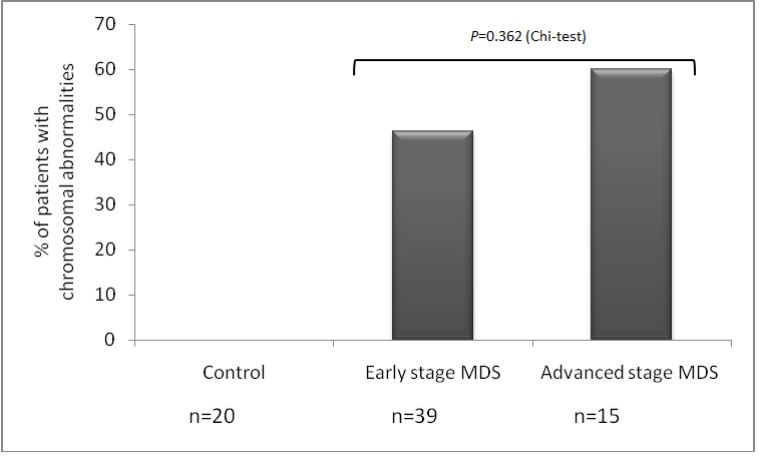
Proportion of patients with chromosome abnormalities identified by conventional karyotyping by patients group. The number of patients with chromosomal aberrations increased from early stage MDS (46.15%) to advanced stage MDS (60%); however, this increase was not statistically significant (P= 0.362).

***HRK*** **promoter hypermethylation in MDS patients**

Methylation curves of standards with known methylation levels were plotted for validation of HRM. The normalized HRM profiles enable the design of MS-HRM for assessment of methylation content of unknown samples with regard to similarities of HRM profiles of standards and unknown. *HRK* hypermethylation was detected by comparing its discriminant methylation state in MDS versus normal samples. Fig. 2 shows an example of the standard curve obtained by HRM method. 

In 11 (20.37%) out of 54 cases, HRK met-hylation (range, 1%-20%) was identified, which was not observed in controls. Average methylation was significantly greater in advanced stage MDS (RAEB-1/RAEB-2) patients compared with early stage MDS (RA/RT/RCMD/5q- syndrome) patients (4.6 vs. 0.025, respectively, P<0.01). Significant difference was observed between RAEB-1 (3 out of 4 cases, 75%), RAEB-2 subgroups (7 out of 11 cases, 63.63%) and RCMD, RA, RT, 5q-syndrome subgroups (P<0.05). Methylation frequency of *HRK* gene in MDS subgroups is indicated in Fig 3A. When patients were classified according to IPSS, methylation level of *HRK* gene in low/Int-1 risk group was statistically lower than that in Int-2/high risk group (0.19 vs. 4.76, respectively, P= 0.00) (Fig. 3B). The methylation level of MDS patients showed a stepwise increase with higher IPSS risk categories. Patients with abnormal karyotype displayed higher methylation levels than patients with normal karyotype. However, the difference was not statistically significant (P=0.302). The frequency of hypermethylated *HRK* gene was statistically different between good and poor cytogenetic risk groups (P<0.01). Study of correlation between promoter methylation status of *HRK* and clinicopathological characteristics is summarized in Table 1. The proportion of patients with chromosomal aberrations detected by standard karyotyping increased from early stage MDS (46.15%) to advanced stage MDS (60%); but not to a statistically significant extent (P=0.362) (Fig. 4). The SF (P<0.01) and LDH levels (P<0.05) in MDS patients were significantly higher compared with healthy subjects and hematologic factors were significantly lower in patient group. Difference in LDH level was significant between advanced stage MDS and early stage MDS patients (*P*=0.002). Increase in LDH level (P=0.006), age (P=0.016) and blast count (P=0.00) was observed in high-risk group, which was significantly higher compared to low-risk group. There was significant correlation between IPSS-R prognostic risk categories and Hb, ANC, Plt count and SF and LDH levels (P<0.05). The LDH level was significantly higher in patients with >5% blasts than group with <5% blasts (P<0.01). There was no difference in SF and LDH levels and hematologic parameters between patients with normal and abnormal karyotype.

## Discussion

MDS is a heterogeneous disease characterized by cytopenia, BM hyperplasia and leukemic transformation (1, 2). HRK is an important pro-apoptotic mitochondrial protein of the BH3-family to interact with the anti-apoptotic proteins to promote apoptosis. Although* HRK* promoter hypermeth-ylation was reported previously in solid tumors, there are no reports on *HRK* methylation and expression in MDS (12, 19-21). Aberrant methylation is a specific characteristic of *HRK* in various tumor cells that is not seen in other BH3- only family genes (BAD, BID, and PUMA) examined (19). Abnormal methylation of *HRK* promoter prevented binding of AP-2α and resulted in down regulation of *HRK* expression, which was followed by resistance to apoptosis and enhanced tumor growth (15). Therefore, HRK might be used as a target for demethylation therapy to stimulate apoptosis in cancer cells (19).

Using MS-HRM, we showed abnormal promoter methylation in *HRK* gene in MDS cells as compared with control cells. We then examined if the methylation status of *HRK* predicted clinical outcomes. Our data from patients with MDS support the idea that aberrant methylation is a progressive process, found in low-risk MDS and significantly increased with more advanced disease. In advanced stage MDS, CpG sites were hypermethylated in more than 60% of patients. Aberrant methylation level was significantly associated with advanced-stage MDS and higher IPSS score. The association of gene methylation with clinicopathological parameters is in favor of the hypothesis that aberrant methylation plays a pathogenic role in MDS and AML evolution. In addition, aberrant DNA methylation and chromosomal aberrations correlated to a statistically significant extent (P=0.001). In this study, trends were detected for an association between presence of methylation on the *HRK* gene in patients with poor cytogenetic subgroup. Therefore, aberrant promoter methylation and cytogenetic abnormalities may cooperate to determine clinical outcome. Jiang et al. investigated average methylation and the number of aberrantly methylated CpG sites in patients with early stage MDS, advanced-stage MDS and AML using DNA methylation microarrays. They found that average methylation and the number of aberrantly methylated CpG sites were significantly higher in the advanced disease. Moreover, they concluded that aberrant DNA methylation is the main mechanism for down regulation of tumor suppressor genes and clonal variation in leukemic transformation (6). In further analysis, methylation status of HRK showed that HRK- associated CpG site methylation was significantly associated with decreased HRK trans-cript levels, which was consistent with findings of other authors (22-25). In addition, decreased gene expression was observed in some patients without methylation, which showss the role of other mechanisms in *HRK* gene silencing. This gene is located at 12q13.1, where gene deletion occurs frequently. Higuchi et al. investigated the relationship of 12q13.1 LOH and methylation with *HRK* expression and its protein levels in prostate cancer. They observed 12q13.1 LOH and hypermethylation in 23% and 38% of their cases, respectively. They concluded that promoter methylation along with LOH were the major mechanisms involved in *HRK* suppression (20-26) . Several prognostic factors for patients with MDS have been identified, including LDH and SF levels. However, their predictive value remains unclear due to AML progression. Our results showed that median LDH level at diagnosis was 410.17 U/L (range 99-1886 U/L). The LDH level in patients with high risk MDS (high risk and intermediate 2), advanced stage MDS (RAEB-1 and RAEB-2) and poor karyotype was obviously increased in comparison to their corresponding groups. In our patients, LDH concentration significantly correlated with methylation in *HRK* gene and IPSS-R risk groups. Elevated LDH at diagnosis may be associated with an increased probability of disease progression. Other studies showed that a serum LDH activity of ≥300 U/L in MDS is associated with a significantly shorter survival and higher risk of AML transformation (27-28). In addition, it has been shown that a high SF at diagnosis is a poor prognostic factor for overall survival in MDS and a baseline SF level could be an indicator for prediction of leukemic evolution in MDS (29). In our analysis, higher-risk MDS was significantly associated with higher SF level.

In summary, the data reported here show that* HRK* proapoptotic gene is suppressed by aberrant methylation of its promoter. We further suggest that patients with *HRK* methylation have a higher incidence of poor karyotype, advanced-stage MDS and higher IPSS-R score than patients without methylation. Defective expression of *HRK* may contribute to progression of MDS. Moreover, the data presented here indicate that LDH and SF are both significant prognostic factors that may be used for evaluation of prognosis in MDS patients.
